# Comparison between central and automated peripheral blood pressure measurement for early detection of kidney dysfunction in hypertensive patients

**DOI:** 10.1186/s12872-023-03292-x

**Published:** 2023-05-17

**Authors:** Ahmed Abdel-Galeel, Nader N. Fawzy, Wageeh A. Ali, Doaa A. Fouad

**Affiliations:** 1 Cardiovascular Medicine Department, Assiut University Heart Hospital, Assiut University, Assiut, Egypt; 2 Radiodiagnosis Department, Assiut University Hospital, Assiut University, Assiut, Egypt

**Keywords:** Hypertension, Chronic kidney disease, Central blood pressure

## Abstract

**Background:**

There is a close relationship between blood pressure levels and the risk of cardiovascular events, strokes, and kidney disease. For many years, the gold standard instrument for blood pressure measurement was a mercury sphygmomanometer and a stethoscope, but this century-old technique of Riva-Rocci/Korotkov is being progressively removed from clinical practice. Central blood pressure is considered better than peripheral blood pressure in predicting cardiovascular events, as it assesses wave reflections and viscoelastic properties of the arterial wall which make systolic and pulse pressures vary from central to peripheral arteries, but mean blood pressure is constant in the conduit arteries.

**Methods:**

The study included 201 patients with primary hypertension (108 patients with chronic kidney disease and 93 patients without kidney disease). All patients underwent blood pressure measurement by OMRON M2 and Mobil-O-Graph devices, kidney function assessment and abdominal ultrasonography.

**Results:**

Patients with chronic kidney disease were significantly older (60.02 ± 9.1 vs. 55.33 ± 8.5; P < 0.001), with longer duration of hypertension (7.56 ± 5.9 vs. 6.05 ± 5.8; P = 0.020) in comparison to those without chronic kidney disease. Automated peripheral measurement of systolic blood pressure, diastolic blood pressure and pulse pressure were significantly higher in comparison to central blood pressure. Patients with chronic kidney disease had significantly higher augmentation index (24.06 ± 12.6 vs. 19.02 ± 10.8; P < 0.001) and pulsed wave velocity (8.66 ± 1.5 vs. 8.69 ± 6.8; P = 0.004) in comparison to those without chronic kidney disease. Augmentation index had positive correlation with pulse wave velocity (r = 0.183, P = 0.005). There was negative correlation between both pulse wave velocity and augmentation index and estimated glomerular filtration rate (r = -0.318, P < 0.001), and (r = -0.236, P < 0.001), respectively. Hence, arterial stiffness parameters are good positive test for prediction of chronic kidney disease.

**Conclusion:**

There is a strong agreement between non-invasive centrally and automated peripherally measured blood pressure in diagnosis of hypertension. But non-invasive central measurements are preferred over automated measurements for early prediction and detection of renal impairment.

## Introduction

Hypertension (HTN) is well known as a silent killer, there is a close relationship between blood pressure levels and the risk of cardiovascular events, strokes, and kidney disease. The prevalence of chronic kidney disease (CKD) is higher in hypertensive patients than in normotensive subjects [1]. CKD is a condition characterized by evidence of renal damage or dysfunction, along with an overwhelming risk of cardiovascular disease [2]. CKD is classified based on estimated glomerular filtration rate (eGFR) and urinary albumin excretion rate (AER) [3]. Clinicians search for markers of renal damage (e.g., abnormalities of urinary sediment or organ structure) to diagnose CKD in patients with eGFR of < 60 mL/min/1.73 m^2^. Diabetes and hypertension are responsible for up to two-thirds of CKD; less frequent causes include glomerulonephritis, nephrolithiasis, and polycystic kidney disease. [3]

For long time, the standard instrument for blood pressure (BP) measurement was a mercury sphygmomanometer and a stethoscope, but this old technique is being progressively removed from clinical practice due to the mercury toxicity and the number of faults that may affect this method [4, 5]. Several other techniques have been developed during the last decade in order to gradually replace the traditional method [6, 7], such as automatic devices using algorithms based on the oscillometric technique. These automated devices have been successfully validated using established protocols, mostly on the general population [8, 9]. Central BP is considered better than peripheral BP in predicting cardiovascular events, as it assesses wave reflections and viscoelastic properties of the arterial wall which make systolic and pulse pressures to vary from central to peripheral arteries but mean blood pressure is constant in the conduit arteries [10].

Several parameters were derived from central BP measurement that helped in prediction of adverse cardiovascular outcomes among hypertensive patients, such as augmentation index and pulse wave velocity (PWV). In the present study, we attempted to assess the difference of blood pressure measurements using central and automated peripheral blood pressure measurement (oscillometric) devices among patients with primary hypertension for early detection of kidney dysfunction and the validity of automated BP measurement device compared with non-invasive central BP measurement device for diagnosis of hypertension. Also, we tried to link between parameters such as augmentation index and pulse wave velocity index and early kidney dysfunction markers.

## Patients and methods

### Study design and population

This is a case comparison study design conducted in Assiut University Heart Hospital between May 2018 and September 2019 and included 201 patients with primary hypertension. All patients with primary hypertension (according to the recent ESC guidelines 2018), with age range between 40 and 70 years old, either on anti-hypertensive medications or not were included. Exclusion criteria were patients presenting with secondary hypertension, diabetes mellitus (DM), end stage renal disease (ESRD) on regular dialysis and patients with collagen diseases. We excluded patients younger than 40 years old due to a high propensity of having secondary hypertension. Also, patients older than 70 years old were excluded as well due to the marked effect of vessel wall on the BP value and other parameters such as augmentation index and PWV.

All patients underwent an initial clinical evaluation, including history taking focusing on age, sex, smoking, anti-hypertensive treatment, duration of HTN and history of previous illness such as DM, collagen disease and CKD (defined as abnormalities of kidney structure or function, with implications for health) [3]. Body mass index (BMI) was calculated for every patient according to the following formula: BMI = Weight in Kgs/Height in m^2^.

### Blood pressure assessment

#### Non-invasive central BP measurement

Central blood pressure monitoring was done non-invasively using a well calibrated device; Mobil-O-Graph (cuff-based oscillometry at the brachial artery) device with inbuilt ARC solver (Austrian Institute of Technology, Vienna, Austria) utilized the technique of pulse wave analysis where pressure waveforms were recorded from peripheral arteries [11, 12]. The non-invasive assessment of estimated PWV and augmentation index were performed in a quiet, temperature-controlled examination room with three measurements were taken with a two-minutes break between them while the patient in a sitting position and using an adequately sized cuff.

We used algorithms to obtain conventional blood pressure readings from brachial systolic and diastolic pressures (central aortic pressure derived either using a generalized transfer function, identification of the late systolic shoulder of the peripheral pressure waveform, or a proprietary algorithm) [13]. In the second step, the brachial cuff is inflated to the diastolic blood pressure level and held for about ten seconds to record the pulse waves. Subsequently, central pressure curves obtained through a transfer function from the peripheral reading are plotted. To measure PWV, the ARC solver method utilizes several parameters from pulse wave analysis and wave separation analysis combined in a proprietary mathematical model, whereby the major determinants are age, central pressure, and aortic characteristic impedance [4, 5]. The following parameters were derived from central pulse wave analysis: (i) Central SBP, (ii) Central PP, (iii) PP amplification [brachial PP/central PP], which consists of the amplification ratio of the PP from central to peripheral arteries, (iv) Augmented pressure [central SBP – forward wave pressure], representing the magnitude of the reflected wave and (v) Augmentation index [(augmented pressure/central PP) − 100], which represents the additional load to which the left ventricle is subjected because of backward wave reflection and depends on the cardiac cycle (thus heart rate), the velocity of the pulse wave and the amplitude of the reflected wave. [14]

#### Automated peripheral BP measurement

Digital measurements of peripheral BP were done by the automatic BP monitor (Oscillometric) named the OMRON M2 basic® device, provided by OMRON Healthcare company, Kyoto, Japan.

The patient seated comfortable at the table in a quiet room and relaxed for about 5–10 min before measurement with their arm at heart level rested on a table with the palm facing up. Wrap the cuff around the upper arm, about 2–3 cm above the elbow. This monitor uses inflation by Fuzzy-Logic controlled by electric pump and an automatic rapid pressure release valve for the deflation, its cuff allows BP measurements in arm circumference of 22–42 cm. The device measure BP and pulse rate with a pressure range of 0-299 mmHg and pulse rate range of 40–180 beats/min. The measurement was repeated three times with a two-minute break between them. The mean of them was recorded then the cuff was removed.

### Laboratory investigations

Five milliliters of venous blood was withdrawn under aseptic condition from patients on presentation for renal function tests including: serum creatinine (normal range is 0.6 to 1.2 mg/dl for the adult male and 0.5 to 1.1 mg/dl for the adult female, by the enzymatic method) and blood urea (normal range is 5 to 20 mg/dl per liter) [15]. GFR (ml/min/1.73 m^2^) was estimated by Cock croft-Gault method [16] and CKD was categorized according to eGFR. Estimated GFR less than 60 is documented to be CKD [3]. Moreover, urine analysis was done to all participants to exclude the presence of albuminuria using dipstick method and albuminuria was categorized among CKD patients according to albumin creatinine ratio (ACR) and albumin excretion rate (AER) [3].

### Abdominal ultrasonography

A trained radiologist examined all patients by 2D abdominal ultrasound using GE VIVDE S5 ultrasound system device to assess kidney echogenicity as a sign of CKD. Also, size, symmetry, cortical thickness, and presence of renal tract obstruction were assessed for possibility of exclusion.

**Collectively, CKD was defined as the presence of one of the following parameters for more than three months as derived by abd.US or lab. investigations.** [3]

- Decrease GFR < 60 ml/min/1.73 m².
- Albuminuria (AER > 30 mg/24 h or ACR > 30 mg/g).
- Kidney damage, as defined by structural abnormalities.

### End points of the study

The primary end point of the study was the detection of early kidney dysfunction among patients with primary hypertension. Additional secondary hypotheses compared between blood pressure monitored centrally and peripherally among primary hypertensive patients.

### Ethical consideration

Informed written consents were obtained from all participants after illustrating all steps of the study. The study was approved by the institutional ethical committee under the IRB number 17,101,738 on 05.04.2018.

### Statistical analysis

Data were verified, coded by the researcher, and analyzed using IBM-SPSS 21.0 (IBM-SPSS Inc., Chicago, IL, USA). Continuous data were expressed in the form of mean ± SD or median (interquartile range) according to their distribution while nominal data were expressed in the form of frequency (percentage). Chi square test was used to compare the difference in distribution of frequencies among different groups. Paired Sample t-test was used to compare the means of BP measurements. Mann-Whitney U test analysis was carried out to compare the medians of dichotomous data that did not follow the normal distribution. Receiver-operating characteristic (ROC) curve was depicted to investigate the diagnostic performance of blood pressure measurements and arterial stiffness parameters for prediction of kidney affection, analyzed as area under the curve (AUC), standard error (SE) and 95% confidence interval (CI). Validity statistics (sensitivity, specificity, positive and negative predictive value) were calculated. Agreement between various blood pressure measurement modalities was examined using Cohen’s kappa coefficient (κ). A significant p value was considered when it is equal to or less than 0.05.

### Sample size calculation

G Power program was used to calculate sample size to detect a significant difference in mean of systolic blood pressure between two groups under the study (early kidney dysfunction and normal kidney function) among primary hypertensive patients. Hypothesized effect size 0.5, alpha error 0.05, power 0.80, allocation ratio 1:1. At least 80 patients should be included in each group with a total sample size 160 patients.

## Results

During the study period, 201 patients with primary hypertension were recruited from Assiut University Heart Hospital. According to the presence of CKD, the study population was divided into two groups:

1. Group I: It included 108 with CKD.
2. Group II: It included 93 patients without CKD.

### Baseline data of study population

Baseline patient characteristics categorized by diagnosis were presented in Table 1. The mean age of enrolled patients was 57.58 ± 9.1 years with range 40–70 years old. More than half (57.7%) of them were males and 83 patients (41.3%) were smokers. Mean age of patients with CKD was significantly higher than those without CKD (60.02 ± 9.1 vs. 55.33 ± 8.5 (years); P < 0.001) with insignificant difference among both groups regarding sex, smoking and BMI. Mean duration of HTN among patients with CKD was significantly higher than among those without CKD (7.56 ± 5.9 vs. 6.05 ± 5.8; P = 0.020). As regards anti-hypertensive therapy, the frequent use of RAAS-inhibitor-containing medications was associated with preserved kidney function, p value 0.001. BP measurement didn’t differ significantly between the two study groups either measured centrally or peripherally, Table 1. 44 Patients (40.7%) with CKD had normal ACR. Regarding ultrasound findings, out of 108 patients with CKD, only 17 patients (15.7%) and 13 patients (12%) had echogenic kidney grade I and II respectively.

**Table 1:** Baseline data of studied patients

Variable	Total (n = 201)	CKD (n = 108)	Non- CKD (n = 93)	P-value
**Age in years (mean ± SD)**	57.58 ± 9.1	60.02 ± 9.1	55.33 ± 8.5	**< 0.001**
**Sex**; male (%)	116 (57.7)	63 (58.3)	53 (57)	0.847
**Smoking (%)**	83 (41.3)	41 (38)	42 (45.2)	0.301
**Duration of HTN in years (mean ± SD)**	6.87 ± 5.1	7.56 ± 5.9	6.05 ± 5.8	**0.020**
**Anti-HTN Treatment**:				**0.001**
**RAAS-containing (%)**	116 (57.7)	48 (46.6)	68 (69.4)	
**Non RAAS-containing (%)**	85 (42.3)	55 (53.4)	30 (30.6)	
**Central SBP (mean ± SD)**	112.74 ± 18.83	112.28 ± 21.22	113.22 ± 16.03	0.72
**Central DBP (mean ± SD)**	76.17 ± 14.61	75.91 ± 16.11	76.44 ± 12.94	0.79
**Central pulse pressure (mean ± SD)**	36.96 ± 10.91	36.5 ± 11.19	37.44 ± 10.64	0.54
**Automated SBP (mean ± SD)**	127.58 ± 20.86	126.04 ± 22.74	129.2 ± 18.65	0.28
**Automated DBP (mean ± SD)**	76.55 ± 13.33	76.9 ± 14.32	76.18 ± 12.26	0.70
**Automated pulse pressure (mean ± SD)**	50.8 ± 15.47	49.02 ± 14.67	52.67 ± 16.13	0.09
**Augmentation index**	21.73 ± 12.0	24.06 ± 12.6	19.02 ± 10.8	**< 0.001**
**PWV**	8.86 ± 4.7	8.66 ± 1.5	8.69 ± 6.8	**0.004**
**BMI (Kg/m2) (mean ± SD)**	28.84 ± 4.5	29.16 ± 4.7	28.46 ± 4.2	0.268

Data expressed in the form of the mean and (SD), frequency (percentage). P-value was significant if < 0.05. **CKD**: Chronic kidney disease; **RAAS**: Renin-angiotensin-aldosterone system; **BMI**: Body mass index; **SBP**: systolic blood pressure; **DBP**: diastolic blood pressure; **PWV**: Pulse wave velocity; **ACR**: Albumin creatinine ratio; **e-GFR**: Estimated glomerular filtration rate; **U/S**: Ultrasound examination.

### Blood pressure measurement data of studied patients with different methods

Automatic peripheral measurements of systolic BP, diastolic BP and pulse pressure were significantly higher compared with the central measurements (P < 0.001, 0.044 and  < 0.001) respectively, Table 2.

**Table 2:** Blood pressure measurement data of studied patients with different methods

Variable (n = 201)	Mean ± SD	P-value
**Systolic BP**		
• Central BP	112.74 ± 18.8	**< 0.001**
• Automatic peripheral BP	127.85 ± 20.9	
**Diastolic BP**		
• Central BP	76.17 ± 14.6	**0.044**
• Automatic peripheral BP	76.55 ± 13.3	
**Pulse Pressure**		
• Central pulse pressure	39.96 ± 10.9	**< 0.001**
• Automatic pulse pressure	50.80 ± 15.5	

Data expressed in the form of the mean and (SD). P-value was significant if < 0.05. **BP**: blood pressure.

### Diagnostic performance of automatic peripheral versus central BP measurements in the diagnosis of HTN

**Fig. 1:**
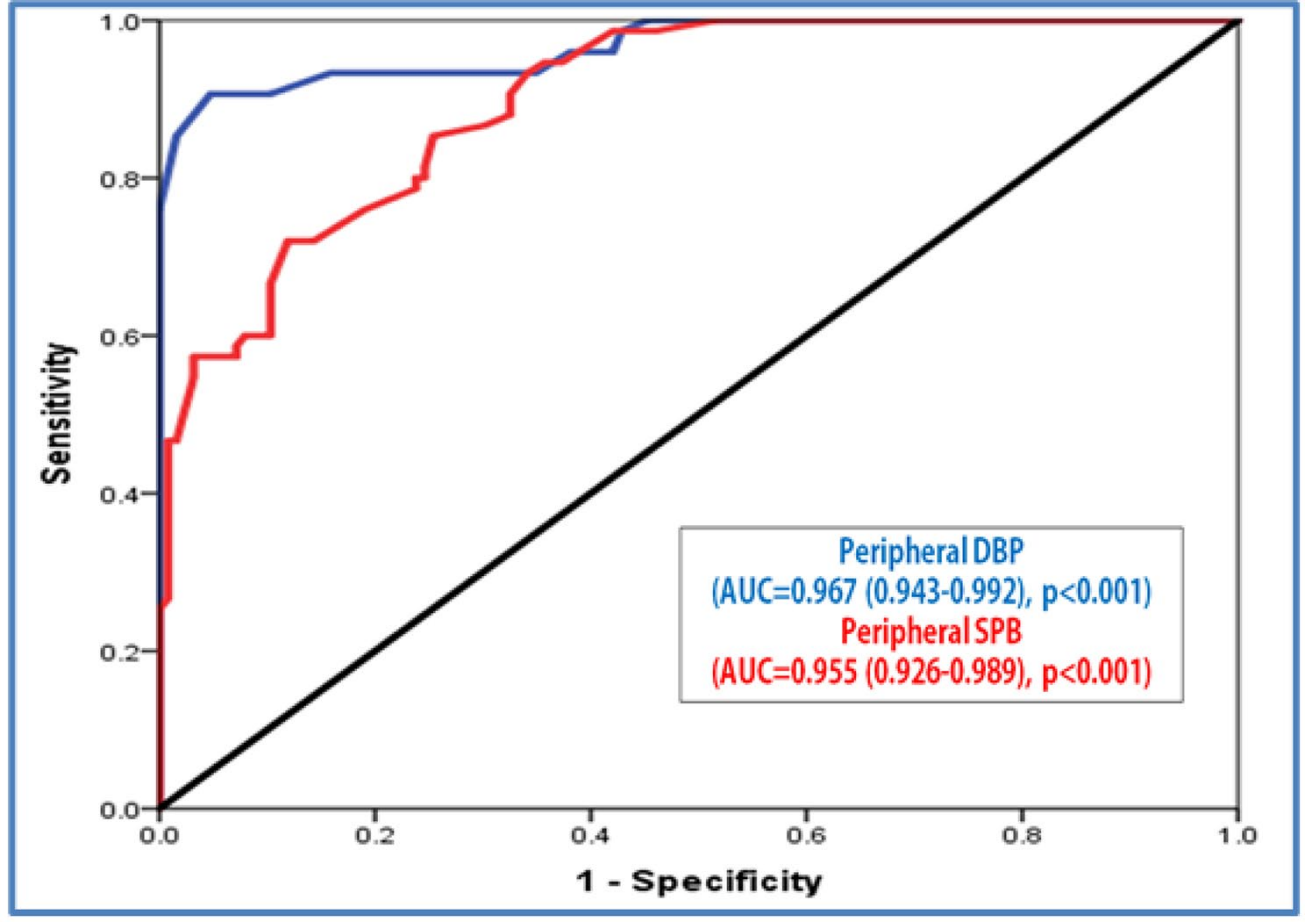
ROC curve for validity of automatic peripheral BP measurements according to central BP in the diagnosis of HTN.

To test the validity of automatic peripheral BP measurement in comparison to central BP measurements in the diagnosis of HTN, ROC curves were plotted. Figure 1 showed agreement between central and automatic peripheral measurement of BP with AUC of 0.967 and 0.955 for DBP and SBP, respectively (P < 0.001) with 91% and 94% sensitivity, and 96% and 100% specificity for both systolic and diastolic BP respectively in diagnosis of HTN.

### Diagnostic performance of BP measurements for prediction of CKD

Our results reported that various BP measurements values didn’t have any significance for prediction of CKD. Hence, all these measurements couldn’t be used for prediction of CKD among patients with HTN.

### Parameters of arterial stiffness among studied patients

Patients with CKD had significantly higher augmentation index (P < 0.001) and PWV (P = 0.004) in comparison to those without CKD as shown in Table 2. Moreover, augmentation index had a positive correlation with PWV among all studied patients (r = 0.183, P = 0.005) and among CKD patients (r = 0.233, P = 0.009). This correlation was weak and statistically insignificant among non-CKD patients (r = 0.018, P = 0.430).

### Correlation between arterial stiffness parameters and e-GFR

PWV had a negative correlation with e-GFR either among all studied group, non-CKD group and CKD group (r = -0.318, -0.471, and − 0.171, P < 0.001, < 0.001 and 0.043 respectively). While augmentation index showed a negative correlation with e-GFR among only all studied group (r = -0.236, P < 0.001). This relation was lost among non-CKD and CKD patients, Table 3.

**Table 3:** Correlation between arterial stiffness parameters and e-GFR.

	e-GFR	
	r	P-value
**All Patients (n = 201)**		
• PWV	-0.318	**< 0.001**
• Augmentation	-0.236	**< 0.001**
**Non-CKD (n = 93)**		
• PWV	-0.471	**< 0.001**
• Augmentation	-0.039	= 0.351
**CKD (n = 108)**		
• PWV	-0.171	**= 0.043**
• Augmentation	-0.003	= 0.488

Data expressed in the form of r value (strength of correlation), P-value (significance of correlation and considered significant if < 0.05). **CKD** = chronic kidney disease, **e-GFR** = Estimated globular filtration rate, **PWV** = Pulse wave velocity.

### Relationship between arterial stiffness parameters and CKD parameters among group I (CKD patients)

Patients with normal ACR had significantly lower PWV (P = 0.002) and augmentation index (P = 0.049) in comparison to those with abnormal ACR. Also, patients with normal abdominal ultrasound had significantly lower PWV in comparison to those with echogenic kidney grade I (8.67 ± 5.1 vs. 8.80 ± 1.3) and grade II (8.67 ± 5.1 vs. 8.82 ± 1.6). Augmentation index was significantly lower among patients with normal abdominal ultrasound in comparison to those with echogenic kidney grade I (21.46 ± 12.1 vs. 21.56 ± 8.6) and grade II (21.46 ± 12.1 vs. 21.62 ± 14.5) as shown in Table 4.

**Table 4:** Relationship between arterial stiffness parameters among CKD patients

Cut-off	PWV 7 m/s		Augmentation Index 10	
	Mean ± SD	Median (Range)	Mean ± SD	Median (Range)
**ACR**				
• Normal	8.62 ± 5.7	8 (5–72)	20.59 ± 11.5	20 (1–53)
• Abnormal	8.81 ± 1.4	9.1 (5.7–11.8)	24.16 ± 12.8	26.5 (3–44)
**P-value**	**= 0.002**		**= 0.049**	
**U/S**				
• Normal	8.67 ± 5.1	8.3 (5–72)	21.46 ± 12.1	22 (1–53)
• G 1	8.80 ± 1.3	9.1 (7–11)	21.56 ± 8.6	22 (7–33)
• G 2	8.82 ± 1.6	9.3 (6–12)	21.62 ± 14.5	31 (9–44)
**P-value**	**= 0.034**		**= 0.044**	

Data expressed in the form of the mean and (SD) and frequency. P-value was significant if < 0.05.**ACR** = Albumin creatinine ratio, **CKD** = Chronic kidney disease, **U/S** = Ultrasound, **PWV** = Pulse wave velocity.

### Diagnostic performance of arterial stiffness parameters for prediction of CKD

**Fig. 2:**
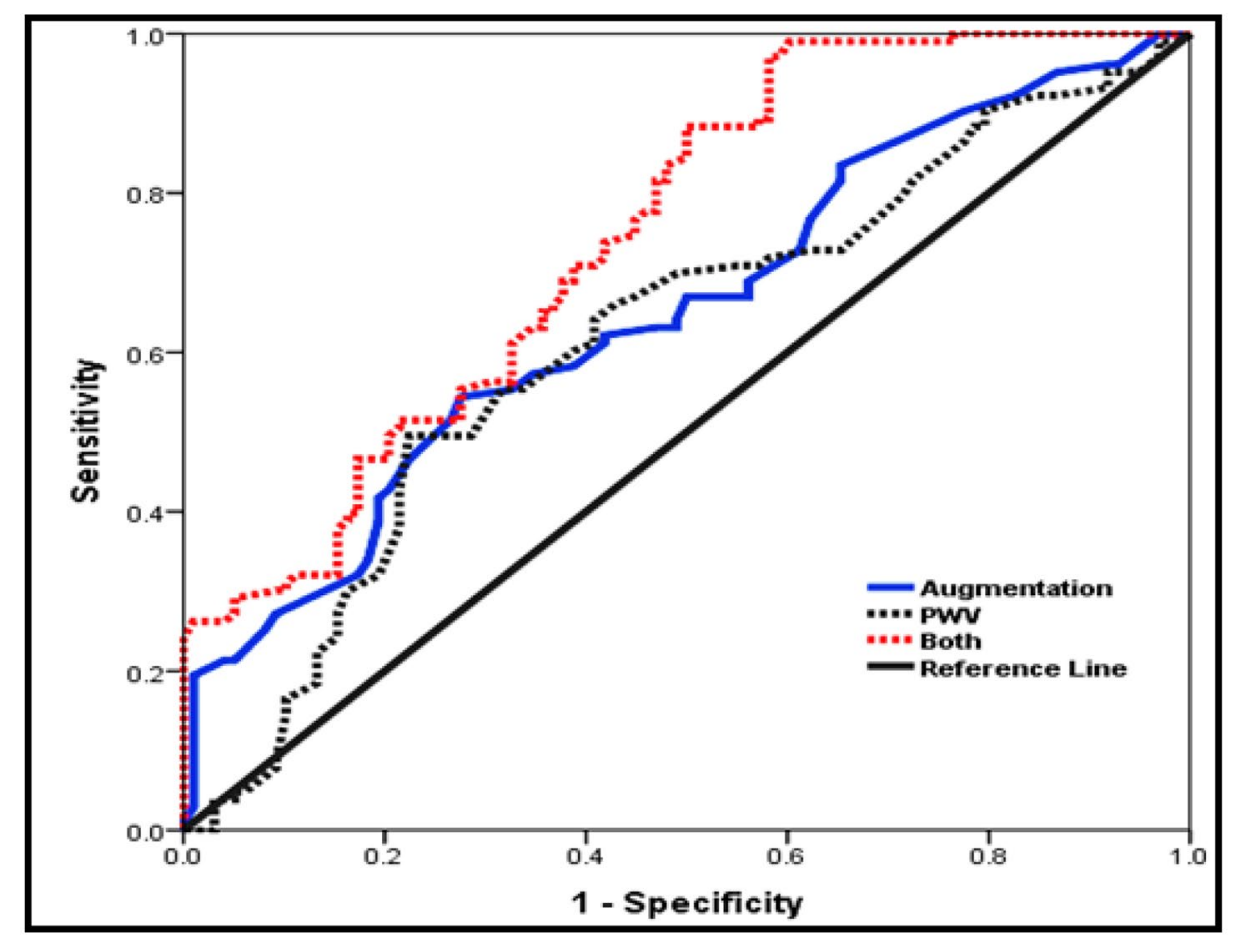
ROC curve for arterial stiffness parameter for CKD Prediction

Figure 2 demonstrated the predictive power of arterial stiffness parameters. It was noticed that augmentation index at cut-off point > 10 had 82% sensitivity and 51% specificity (AUC 0.651, P < 0.001) for prediction of CKD while PWV at cut-off point > 7 m/s, had 84% sensitivity and 45% specificity (AUC 0.617, P = 0.040) for prediction of CKD. Better prediction was revealed for the combined arterial stiffness parameters (augmentation index and PWV). The combined parameters had 94% sensitivity and 65% specificity (AUC 0.772, P < 0.001) for prediction of CKD.

## Discussion

Hypertension is a common problem among older adults, reaching prevalence as high as 70 to 80%. Untreated hypertension is notorious for increasing the risk of mortality and is often described as a silent killer. It may be associated with a risk of atherosclerotic disease among 30% and organ damage among 50% of people within 8–10 years after its onset. End stage renal disease is one of the main complications of HTN particularly among diabetics patients [17, 18].

The current study enrolled 201 hypertensive patients who were classified into 108 patients with CKD and 93 patients without CKD. We aimed to assess two different methods in BP measurement and test their value in prediction of early renal dysfunction among hypertensive patients.

We noticed that patients with CKD had significantly higher mean age and mean duration of HTN in comparison to those without CKD. Also, there was male predominance among CKD group but of no statistical significance. Our results were consistent with Tannor et al. who stated that patients with CKD had significantly higher mean age and longer duration of HTN. Also, they reported that age was considered a predictor for CKD among patients with HTN and male patients had higher risk to progress to CKD in comparison to females [19]. We noticed that usage of ACEIs or ARBs was significantly higher among patients without CKD. This was in concordance with major guideline recommending the use of ACEIs or ARBs for their benefits in decreasing proteinuria and preventing progression of kidney disease [14]. Most enrolled patients were on low or conventional doses of ACEI or ARBs but high doses had been shown to improve proteinuria and prevent CKD progression [20].

Reliability of brachial BP measurement in the physician’s office has its own limitations that make it less reliable than out-of-office BP measurement, using either home BP monitoring or ambulatory BP monitoring (ABPM) techniques [21]. In recent years, there is increasing evidence that central aortic BP might be superior to peripheral BP in the prediction of cardiovascular events. It was found a close association of central BP with target organ damage and cardiovascular risk [22]. Several issues can complicate the measurement of BP among patients with CKD, including an increased likelihood of arrhythmias, arterial stiffness, and masked hypertension. Accurate BP measurement is particularly important in high cardiovascular disease settings such as those that prevail in patients with CKD [22].

BP is traditionally measured at the brachial arteries, so brachial BP measurement is the gold standard method for diagnosis and management of hypertension. However, in many individuals, there are some discrepancies between central and peripheral BP. These discrepancies have long been a common research aspect, and whether central BP is a better clinical surrogate than brachial BP has also been universally debated [23].

Our study revealed that that automatic peripheral BP measurement slightly overestimated SBP and DBP in comparison to central BP measurement with subsequent overestimation of mean BP either among those with CKD or without CKD in consistent with Drawz et al. who found that peripheral measurement of both SBP and mean BP were overestimated due to the consequence of the calibration method [22].Similarly, in a published meta-analysis about the accuracy of non-invasive measurements of BP concluded that these measures could be used to assess BP and the slightly reported errors in BP estimation by these devices are mostly attributable to the errors introduced during calibration [24]. Moreover, our study revealed a strong degree of agreement between both automatic peripheral and central BP measurements in concordance with Rouxinol-Dias et al. who stated that it might be reasonable to use non-invasive brachial BP as an estimate of central BP [25] and many other authors recommended the use of brachial pressure assessment as an estimate of central BP and the diagnosis of HTN [25, 26].

Till now, the diagnosis of hypertension, according to office, home, or ambulatory BP measurements, is currently based on recordings from the brachial arteries. Because of the phenomenon of PP amplification, brachial BP and PP are usually higher than the corresponding readings in the central aorta [27]. However, either by the auscultatory method or automatic oscillometric sphygmomanometers, the non-invasively measured brachial BP and PP are usually lower than the invasively measured intra-arterial readings. Such variability between central monitoring and cuff measurement of BP may be attributed to number of factors, including age, sex, body height, heart rate, medications, and systemic vascular diseases. Besides, noninvasive brachial SBP as a surrogate for central SBP has been shown to have a large random error [27].

On the other hand, our study demonstrated that diagnostic accuracy of central and automatic peripheral BP measurements had no significant value in the prediction of CKD (P > 0.05). It’s known that central BP may reflect the pulsatile load on the heart and large arteries better than brachial BP and more closely associated with end organ damage [28]. In contrast, the current study disagreed with this concept may be due to relatively small sample size and discordant with a systematic review of 58 studies which revealed that a central BP compared with brachial BP seems to be more strongly associated with most of the investigated indices of preclinical organ damage [28].

Recent technological advances have enabled several non-invasive methods to estimate arterial stiffness. Of these non-invasive measurements; higher PWV and augmentation index as indicators of intra-renal hemodynamics, are known to be associated with worse renal function and further, have a predictive value for renal outcomes among patients with essential HTN [25].

Our study corroborates and extends previous findings that patients with CKD had significantly higher augmentation index (P < 0.001) and PWV (P = 0.004) in comparison to those without CKD. Also, both PWV and augmentation had a negative correlation with eGFR and increased among patients with abnormal ACR. This indicates that PWV and augmentation increases with progression of the renal damage. In concordance with Kusunoki et al. who demonstrated that arterial stiffness indices such as PWV and augmentation index as well renal resistive index (RI) increased with increasing severity of CKD stage among hypertensive patients. They also found that PWV is one of the predictors for advanced renal disease in such patients. This could be explained by excessive pulsatile energy transmission into the susceptible renal microvasculature as an important mechanism of progression of kidney damage and renal resistive index was known to be associated with carotid-femoral PWV and abnormal ACR. So, increased RI was associated with more renal damage [29].

It should be noted that augmentation index at cut-off point 10 had 82% sensitivity (AUC = 0.651) while PWV at cut-off point 7 m/s had 84% sensitivity (AUC = 0.617) for prediction of CKD. Furthermore, combination of augmentation index and PWV improved sensitivity to 94% (AUC = 0.772) for prediction of CKD. Overall, our positive results of the combined augmentation index and PWV agreed with Fouad et al., who revealed that PWV was significantly higher among hypertensive patients compared to normotensive patients which make PWV is a good predictor for end organ damage [21].

It is important to note that, our data indicated that PWV and augmentation index provide complementary information on target organ damage among hypertensive patients, but its predictive role for CKD as organ damage development parameter needs to be assessed in large prospective clinical trials. Also, our data reinforced the concept that automatic peripheral BP measurement may be helpful in diagnosing and monitoring hypertension. But some of the normotensives in the office and some of the controlled hypertensive appeared to show some type of masked hypertension, as well as an increase in central pressure, both of which are associated with the increase in aortic stiffness as measured by PWV and augmentation index.

Finally, it has long been recognized that individual discrepancies between central and peripheral BP may be magnified during hemodynamic changes or after pharmacological interventions. The differential responses of central BP vs. brachial BP to various antihypertensive agents are highly variable among individuals in clinical studies [30].

### Study limitations

We acknowledge a few limitations for the present study. First, our study is limited by the enrollment of a relatively small sample included from a single center. However, this was overcome using appropriate statistical tests. The results, therefore, are preliminary and need to be confirmed and extended in larger multicenter studies on a larger number of patients to get more valid and reliable diagnostic impact.

## Conclusion

There is a strong agreement between non-invasive central BP measurements and automated peripheral BP measurements in the diagnosis of HTN. But non-invasive central measurements are preferred over automated measurements for early prediction and detection of renal impairment.

## Data Availability

The data underlying this article will be shared on request with the corresponding author.
